# Supplementary material for: Quantum storage of entangled photons at telecom wavelengths in a crystal
Source: Nat Commun. 2023 Nov 1;14:6995. doi: 10.1038/s41467-023-42741-1 (PMC10620411; doi:10.1038/s41467-023-42741-1)
Supplement: Supplementary file 1 — Supplementary Information [file 41467_2023_42741_MOESM1_ESM.pdf]

Supplementary Information for: Quantum storage of entangled photons at telecom wavelengths in a crystal

Ming-Hao Jiang,[1,*] Wenyi Xue,[1,*] Qian He,[1] Yu-Yang An,[1] Xiaodong Zheng,[1] Wen-Jie Xu,[1] Yu-Bo Xie,[1] Yanqing Lu,[1] Shining Zhu,[1] and Xiao-Song Ma[1,2,3,†]

[1]*National Laboratory of Solid-state Microstructures, School of Physics, College of Engineering and Applied Sciences, Collaborative Innovation Center of Advanced Microstructures, Nanjing University, Nanjing 210093, China*

[2]*Synergetic Innovation Center of Quantum Information and Quantum Physics, University of Science and Technology of China, Hefei, Anhui 230026, China*

[3]*Hefei National Laboratory, Hefei 230088, China*

(Dated: October 16, 2023)

Supplementary Note 1 - Experimental time sequence

The time sequence of the experiment is shown in Supplementary Fig. 1. First, the polarization laser sweep between 550 MHz and 750 MHz blue shifted from the center of the AFC for 1.8 s, partially polarize the ions and increase the optical depth at the AFC's band. Second, the AFC laser is intensity modulated into pulses with a period equal to the storage time t_{M} and a pulse duration of 6 ns. The AFC preparation process continues for 1.9 s. After a 0.2 s delay, the pump laser of the photon source is intensity modulated into pulses with a period of 32 ns and a pulse duration of 4 ns. The generated signal photons are stored in the memory and retrieved for entanglement analysis. This memory window continues for 1 s, followed by another 0.1 s delay, resulting in a 20% duty cycle of the memory.

Supplementary Note 2 – Entangled photon pair source

The chip-integrated silicon nitride (SiN) dual Mach-Zehnder interferometer resonator (DMZI-R) source is fabricated by advanced SiN fabrication technology (Ligentec), which provides ultra-low propagation loss. The fibre polarization controller before the two H48 wavelength division multiplexers (WDM) makes sure the light coupled to the chip is in single polarization mode. The light is coupled into or out from the chip by a V groove fibre array with a pitch of about 127 μm.

Our DMZI-R has a radius of about 230 μm and the free spectral range (FSR) is close to 100 GHz for TM mode. The length difference of the two unbalanced MZIs is about 240 μm and hence the DMZI interference period is about 600 GHz. The gaps between the waveguide to the ring at the In-Through side and Add-Drop side are both nominally 500 nm. The relatively large size of components and the small thermo-optic coefficient of silicon nitride reduce the thermal crosstalk between different thermo-optical phase shifters. The resistors on the In-Through MZI, Add-Drop MZI and the central ring are about 3130, 1350, and 2270 Ohms, respectively. The resistor on the ring, in conjunction with the TEC for the whole chip, tunes the wavelength of the photon pairs to match quantum storage and the relative phase between the different paths of the Add-Drop MZI at the same time.

As discussed in main text, the non-ideal visibilities of Franson interference are mainly due to the high-order photon pairs generated from SFWM process, and the phase fluctuations

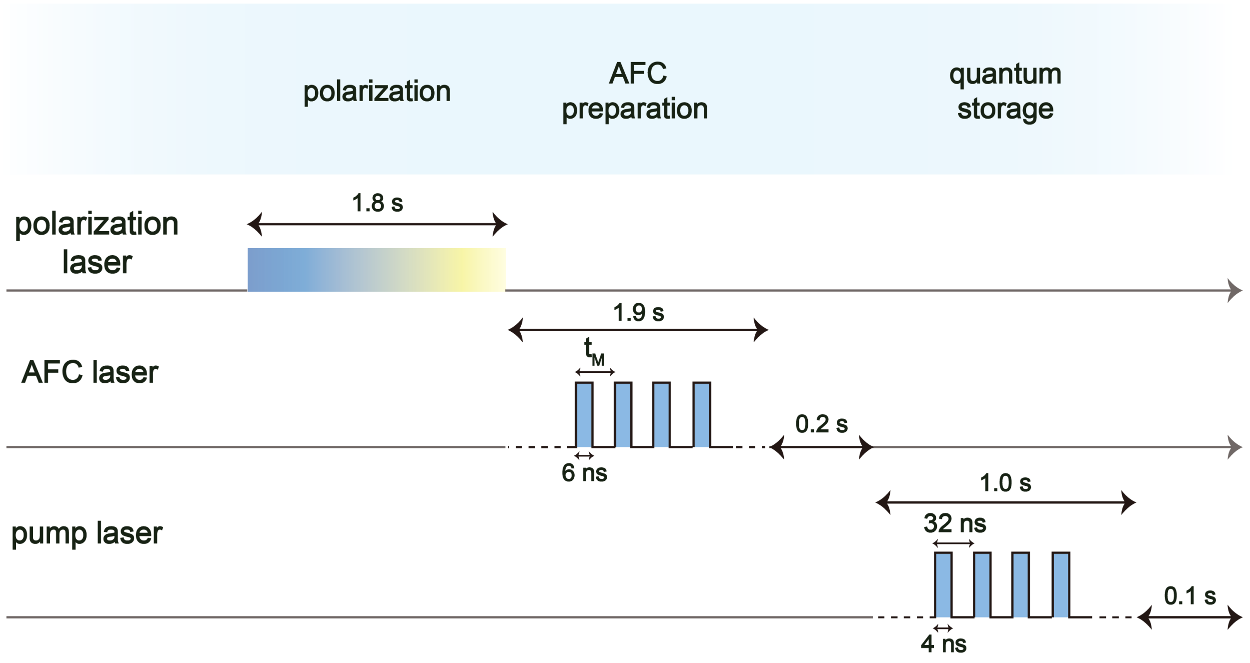

Supplementary figure 1. Experimental time sequence.

of the Franson interferometers. To confirm the high-order photon-pair emission's impact on visibilities, we reduce the average power of the pulsed pump to 1.4 mW, and obtain visibilities of 84.8 ± 0.3% and 86.4 ± 0.3%, as shown in Supplementary Fig. 2.

Supplementary Note 3 – Time correlation of the idler and signal photons

The idler and signal photons are generated simultaneously by a SFWM process, resulting in a defined time correlation. However, the photons are trapped by the MRR cavity and the correlation peak is broadened. The average lifetime of photons in a cavity is given by

$$T_{\mathrm{cav}} = \frac{1}{2\pi\Delta\nu}, \tag{1}$$

where $\Delta\nu$ is the linewidth of the cavity. The probability that a photon escapes from the cavity with a delay of t after its generation is proportional to $e^{-t/T_{\mathrm{cav}}}$. For an idler-start-and-signal-stop detection, the probability of a coincidence with a time difference of τ is given as below:

$$\tau > 0: \quad p \propto \int_0^\infty e^{-\frac{t}{T_{\mathrm{i}}}} e^{-\frac{t+\tau}{T_{\mathrm{s}}}} dt = e^{-\frac{\tau}{T_{\mathrm{s}}}} \int_0^\infty e^{-\frac{t}{T_{\mathrm{i}}}} e^{-\frac{t}{T_{\mathrm{s}}}} dt = e^{-\frac{\tau}{T_{\mathrm{s}}}} \times \mathrm{const}, \tag{2a}$$

$$\tau < 0: \quad p \propto \int_0^\infty e^{-\frac{t-\tau}{T_{\mathrm{i}}}} e^{-\frac{t}{T_{\mathrm{s}}}} dt = e^{\frac{\tau}{T_{\mathrm{i}}}} \int_0^\infty e^{-\frac{t}{T_{\mathrm{i}}}} e^{-\frac{t}{T_{\mathrm{s}}}} dt = e^{\frac{\tau}{T_{\mathrm{i}}}} \times \mathrm{const}, \tag{2b}$$

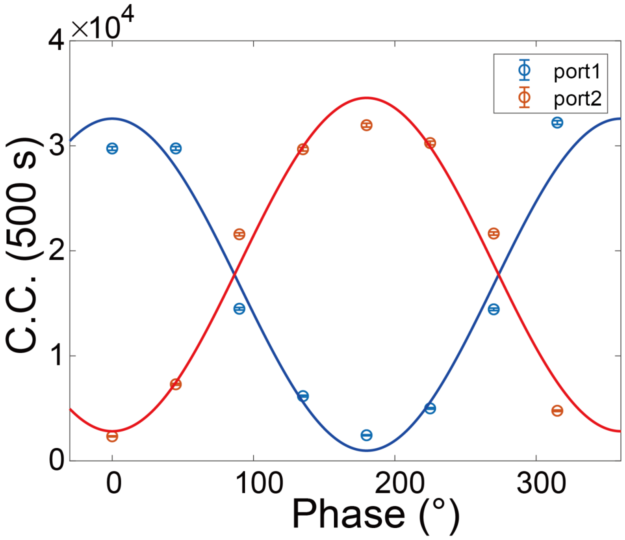

Supplementary figure 2. Franson interference. Two-photon coincidence counts as functions of the phase between two Franson interferometers with 1.4-mW pulsed pump. The blue/red circles are the raw data of coincidence counts between idler and port 1/2 of the signal photon, respectively. Their visibilities are $84.8 \pm 0.3\%$ and $86.4 \pm 0.3\%$. See text for details. Error bars are derived from Poissonian statistics and error propagation.

where T_i and T_s are the cavity lifetime for the idler and signal photons, respectively. By using this model, we obtain the lifetime for the signal and idler photons to be 980 ps and 997 ps respectively, as presented in Fig. 2**c** of the main text.

Supplementary Note 4 – Details of the experimental conditions

A 50 ppm doped $^{167}\mathrm{Er}^{3+}$:$\mathrm{Y_2SiO_5}$ $4\times5\times9$ mm^3 (D_1, D_2, b) crystal is cooled to about 230 mK in a dilution refrigerator integrated with a superconducting magnet. The laser and signal photons are focused on the crystal with a waist of about 40 μm. The temperature of the $^{167}\mathrm{Er}^{3+}$ ensemble is deduced from the thermal-equilibrium population between two ground Zeeman levels at different magnetic fields. The hyperfine states of $^{167}\mathrm{Er}^{3+}$ are not splitted clearly in a low magnetic field. Therefore, we use another 10 ppm doped non-isotopic purified Er^{3+}:$\mathrm{Y_2SiO_5}$ crystal to calibrate the temperature under the similar experiment configuration (such as power and beam size), as shown in Supplementary Fig. 3.

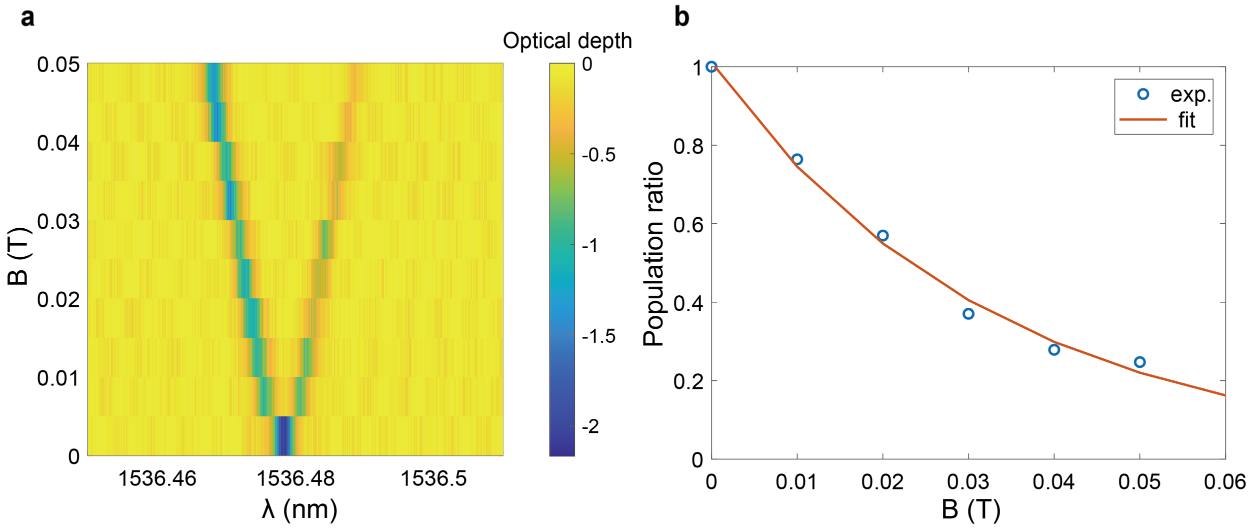

Supplementary figure 3. Temperature estimation. a. Zeeman splitting of the non-isotopic Er^{3+}:Y_2SiO_5 crystal. **b**. The populations ratio between the two Zeeman levels in Supplementary Fig. 3**a** are extracted, and fit to Boltzmann distribution with $T = 230$ mK.

Supplementary Note 5 – Optimization of storage time

As shown in Supplementary Fig. 4, when increasing the magnetic field applied to the crystal, we find 2 side holes splitting from the central hole we burnt. The splitting of the side holes is proportional to the magnetic field (~ 2.11 MHz/T), which is similar to that in Er^{3+}:Ti^{4+}:$LiNbO_3$ waveguide[1] and may also arise from the interactions between the erbium electronic spin and the nuclear spin of Y in the host crystal. With a field of about 1.5 T, the side hole splitting is about 3.17 MHz, corresponding to an AFC spectrum with the storage time of about 315 ns. We optimize the AFC storage time based on: 1. It is half-integer times of 32 ns (time difference of the two arms of the AMZIs), so that the retrieved coincidence peak is at the middle of two accidental peaks (see Fig. 5**a**, **b** of the main text); 2. It is approximately integer times of 315 ns for reducing the side hole spectrum influences. In this way, we realize quantum storage of the entangled photons for up to 1936 μs, as presented in the main text. Note that these side holes may limit optical excited state storage time in $^{167}Er^{3+}$:Y_2SiO_5.

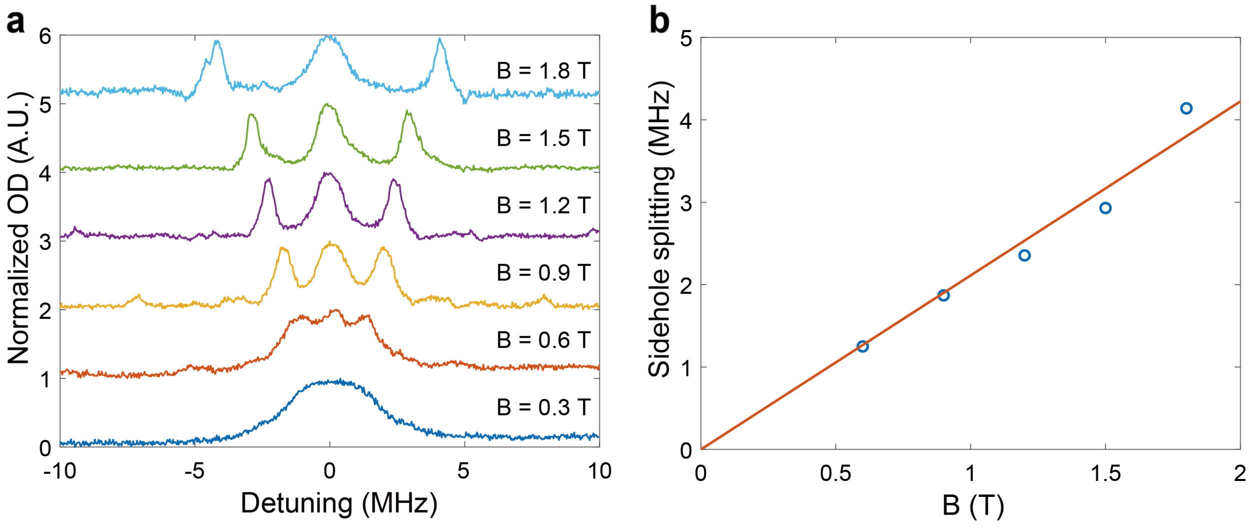

Supplementary figure 4. Side holes in $^{167}Er^{3+}$:Y_2SiO_5**. a**. Two side holes split from the central spectral hole with the magnetic field increased. **b**. Side hole splitting as a function of the magnetic field.

Supplementary Note 6 – Theoretical storage efficiency

In AFC scheme, the theoretical storage efficiency can be calculated from the comb profile as

$$\eta = \frac{d^2}{F^2} e^{-\frac{d}{F}} e^{-d_0} e^{-\frac{1}{F^2}\frac{\pi^2}{2ln2}}, \tag{3}$$

where d and d_0 are the optical depth of AFC and background absorption, and F is the finesse of the AFC[2]. The AFC teeth are considered as Lorentzian peaks here. For a maximal efficiency, the AFC teeth should have a square shape[3], with an optimized half width of

$$\Gamma^{\mathrm{S}}_{\mathrm{OPT}}(d) = \frac{1}{t_{\mathrm{M}}} \arctan(\frac{2\pi}{d}), \tag{4}$$

where t_{M} is the storage time. For low optical depth d, the optimized finesse is F=2. The corresponding storage efficiency is given by

$$\eta^{\mathrm{S}}_{\mathrm{OPT}}(d) = (\frac{d}{\pi})^2 \sin^2(\Gamma^{\mathrm{S}}_{\mathrm{OPT}} t_{\mathrm{M}}) e^{-d\Gamma^{\mathrm{S}}_{\mathrm{OPT}} t_{\mathrm{M}}/\pi}. \tag{5}$$

In this experiment, the optical depth at the AFC's band is about $d = 2.1$, corresponding to a theoretical efficiency of $\eta^{\mathrm{S}}_{\mathrm{OPT}}(d) = 17.4\%$. The background absorption of $d_0 = 0.8$ reduces the efficiency to $\eta^{\mathrm{S}}_{\mathrm{OPT}}(d - d_0) \times e^{-d_0} = 4.2\%$. The maximal efficiency we measured is $\eta = 3.5\%$

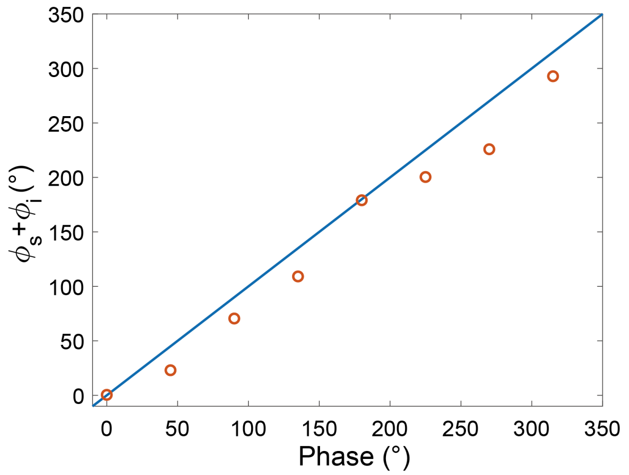

Supplementary figure 5. Phase of the Franson interferometers. The total phases of the pump laser on the two interferometers as a function of the phase setpoint.

for 1296 ns storage. The efficiency can be improved by reducing the background absorption and optimizing the finesse and lineshape of the AFC. Moreover, to further enhance the memory efficiency to unity in AFC protocol, one can use the cavity enhanced scheme[4].

Supplementary Note 7 – Phase lock of the unbalanced interferometers

In this experiment, the time difference of the unbalanced interferometers is much longer than the coherence time of idler and signal photons, eliminating the single photon interference. The Franson interference of coincidence arises from the coherence of the pump laser, which makes it the natural choice for the reference of the Franson interferometers.

The phase of the Franson interferometers is locked with two PID feedback controllers by using the transmission of the pump laser as a reference. With PID controllers, we cannot lock the phase of each interferometer to the top or bottom point, but the total phase of the two interferometers can reach the full 2π period. The phase is determined by the central coincidence peaks of port 1/2 and the relative phase to the maximal/minimal points. The phase of the Franson interference agrees with the sum of the pump laser's phases on the two interferometers, as shown in Supplementary Fig. 5. The output of the photodetectors for different phases are shown in Supplementary Tab. 1, where the sign denotes the positive or negative edge of the PID controllers.

Supplementary table 1. The PID setpoints for the output of the photodetectors and the total phases of the pump laser on the two interferometers.

Phase (°)	U_{idler} (V)	U_{signal} (V)	ϕ_i+ϕ_s (°)
0	+12.2	-15.0	0.4
45	+7.6	-15.0	23.0
90	-20.1	+8.1	70.5
135	+16.5	+25.0	109.1
180	+9.7	+17.2	179.0
225	+8.0	+14.0	200.4
270	+7.0	+9.1	225.9
315	+19.5	-8.0	292.9

Supplementary Note 8 – Calculation of entanglement witness

When the AMZIs are not employed, the quantum state of the photon pair (Eq. (1) in the main text) is projected onto Z basis. The coincidence for $|ee\rangle$ or $|ll\rangle$ states will be at the time difference of 0, while that of the $|el\rangle$ or $|le\rangle$ states will be at the time difference of ±32 ns. Since the cross-correlation is calculated as $g^2_{\text{si}}(0) = \frac{p_{\text{si}}}{p_{\text{s}}p_{\text{i}}} = \frac{\text{C.C.}(\tau=0)}{\text{C.C.}(\tau=\pm 32\text{ns})}$, the probability of projections onto $|el\rangle$ and $|le\rangle$ states (or $|z^+z^-\rangle$ and $|z^-z^+\rangle$ states) can be written as

$$\frac{1}{2}(p(|z^+z^-\rangle) + p(|z^-z^+\rangle)) = \frac{1}{g^2_{\text{si}}(0) + 2}. \tag{6}$$

When the AMZIs are employed, the photon pair is projected onto the X-Y plane. A photon will be projected to the $|x^+\rangle$, $|x^-\rangle$, $|y^+\rangle$ or $|y^-\rangle$ states with its AMZI set to 0, π, $-\pi/2$ or $\pi/2$, respectively. The projections onto $|x^+x^-\rangle$ and $|x^-x^+\rangle$ ($|y^+y^-\rangle$ and $|y^-y^+\rangle$) states will result in a total phase of π (0), which is the fully destructive (constructive) point of the Franson interference. As a result, we will obtain

$$\frac{1}{2}(p(|x^+x^-\rangle) + p(|x^-x^+\rangle)) = \frac{1-V}{4}, \tag{7}$$

$$\frac{1}{2}(p(|y^+y^-\rangle) + p(|y^-y^+\rangle)) = \frac{1+V}{4}, \tag{8}$$

where V is the visibility of the Franson interference.

Therefore, the entanglement witness can be derived from the cross-correlation and visibility of Franson interference as below:

$$\begin{aligned}\langle\Phi|\hat{W}|\Phi\rangle &= \frac{1}{2}(p(|z^+z^-\rangle)+p(|z^-z^+\rangle)+p(|x^+x^-\rangle)+p(|x^-x^+\rangle)-p(|y^+y^-\rangle)-p(|y^-y^+\rangle)) \\ &= \frac{1}{g^2_{\mathrm{si}}(0)+2}-\frac{V}{2}\end{aligned} \tag{9}$$

* These authors contributed equally: Ming-Hao Jiang, Wenyi Xue

† Xiaosong.Ma@nju.edu.cn

[1] M. F. Askarani, M. G. Puigibert, T. Lutz, V. B. Verma, M. D. Shaw, S. W. Nam, N. Sinclair, D. Oblak, and W. Tittel, Storage and reemission of heralded telecommunication-wavelength photons using a crystal waveguide, Phys. Rev. Applied **11**, 054056 (2019).

[2] M. Afzelius, C. Simon, H. de Riedmatten, and N. Gisin, Multimode quantum memory based on atomic frequency combs, Phys. Rev. A **79**, 052329 (2009).

[3] M. Bonarota, J. Ruggiero, J.-L. Le Gouët, and T. Chanelière, Efficiency optimization for atomic frequency comb storage, Phys. Rev. A **81**, 033803 (2010).

[4] M. Afzelius and C. Simon, Impedance-matched cavity quantum memory, Phys. Rev. A **82**, 022310 (2010).
